# Bone Stability After Immediate Implants and Alveolar Ridge Preservation: A 15-Year Retrospective Clinical Study

**DOI:** 10.3390/dj13070299

**Published:** 2025-07-02

**Authors:** Nicola De Angelis, Paolo Pesce, Catherine Yumang, Domenico Baldi, Maria Menini

**Affiliations:** 1 Department of Surgical Sciences and Integrated Diagnostics, Unit of Implant and Prosthodontics, University of Genoa, 16126 Genova, Italy; paolo.pesce@unige.it (P.P.); domanico.baldi@unige.it (D.B.); maria.menini@unige.it (M.M.); 2 Dental Department, University Technology MARA, Sungai Buloh 47000, Malaysia; 3 Private Practice, 15011 Acqui Terme, Italy; drcatyumang06@gmail.com

**Keywords:** tooth extraction, alveolar ridge, bone resorption, immediate implant, bone substitute

## Abstract

**Background:** In modern dentistry, alveolar socket preservation after tooth extraction plays a critical role in maintaining the alveolar ridge for future dental implants. This retrospective clinical study evaluated bone-level changes 15 years after immediate implant placement, coupled with alveolar ridge preservation. **Methods:** Fifty non-smoking patients aged 25 to 75 (30 males and 20 females) who underwent single-implant rehabilitation in both anterior and posterior regions of the upper and lower jaws were included. The study examined bone levels and implant survival over time, using standardized intraoral radiographs at 1, 5, and 15 years post-loading. Implants were placed immediately after atraumatic extraction, and the residual gap was grafted with bovine hydroxyapatite and covered with a collagen membrane. The primary outcome was bone-level stability, while secondary outcomes included implant failure. No temporary crowns or removable dentures were provided during healing. Radiographs were digitized for detailed analysis. **Results:** The results for 50 patients with immediate implant placement showed that bone-resorption levels were significantly higher in the upper jaw than in the lower jaw. **Conclusions:** Posterior implants exhibited greater bone loss than anterior implants, particularly at 1 year and 15 years, while no implant failures occurred.

## 1. Introduction

In the rapidly evolving discipline of contemporary dentistry, the significance of alveolar socket conservation after tooth removal has become more widely acknowledged. This method, which incorporates various strategies, is essential for establishing favorable conditions for future dental implant procedures [1]. Alveolar ridge maintenance is not solely about preserving bone formations; it also encompasses a comprehensive strategy aimed at conserving both hard and soft tissues in the extraction site [2]. A prevalent question among practitioners is whether to insert an immediate implant post-extraction and concurrently fill the residual gap with bone substitutes and absorbable membranes or alternatively to leave the gap unfilled after implant placement, or as another option, to delay implant placement until bone healing occurs. Recent comprehensive reviews have attempted to resolve this clinical question. Seyssens et al. [3] performed a comprehensive review to evaluate the effectiveness of socket grafting and immediate implant insertion. The outcomes reviewed included changes in midfacial tissues and the vertical dimension of the bone around the implants. The review concluded that socket grafting might support horizontal bone conservation and soft tissue stability at the midfacial region of immediate implants, recommending it as an adjunct to immediate implant placement in practice.

Another clinical consideration pertains to the location of implant placement. Although immediate implant placement is frequent for anterior teeth, the literature continues to debate its suitability in posterior sites. A recent systematic review [4] examined implant survival and aesthetic outcomes, assessed by the pink aesthetic score (PES), as primary outcomes; and peri-implant bone loss and implant complications across different clinical scenarios (immediate implant placement, delayed implant placement, and socket preservation) as secondary outcomes. The subgroup analysis indicated that the anterior region showed superior results with immediate implants, whereas the molar region showed better outcomes with delayed implants. The quantitative analysis revealed no significant difference in peri-implant bone loss between immediate and delayed implant protocols (*p* = 0.42).

Various materials are available for filling the residual gap, including xenografts; allografts; and synthetic compounds, like hydroxyapatite and beta tricalcium phosphate [5,6,7]. However, no single choice is universally recommended by most evidence, leaving the decision to the clinician’s expertise [8,9,10]. The same conclusion applies to the sealing systems, which may utilize resorbable or non-resorbable membranes [11,12,13] or, more recently, 3D-printed polymeric materials.

Moreover, although several studies report successful outcomes for post-extractive implants, the literature still lacks long-term evaluations of marginal bone stability around implants placed in extraction sockets, with or without bone grafting. To address this issue, the objective of the present clinical retrospective study is to examine bone levels 15 years after socket grafting and simultaneous implant placement.

## 2. Materials and Methods

### 2.1. Sample Size Calculation

Sample size was calculated as follows:

n = (Z_α/2_ + Z_β_) ^2^ X (2X σ^2^)/ (M_1_ − M_2_)^2^

- **n**: dimension of the group.
- **Z _α/2_**: normal quantile for the chosen significance level (5%).
- **Z _β_**_:_ normal quantile for the desired power (80%).
- **σ^2^**: variance of the variable (marginal bone level) (0.2).
- **M1 − M2**: minimum significant difference between the means of the two groups.

A total of 50 patients were requested for the retrospective analysis, and all the data of the equation above were retrieved in the context of clinical trials and epidemiological studies on ClinCalc and other medical research platforms.

A further post hoc power analysis was calculated with *t*-test on 50 subjects with an expected difference between the groups of 1 mm (marginal bone levels), a standard deviation of 0.2 mm, and a level of significance α of 0.05, with a 1.0 result (100%).

### 2.2. Methods

This retrospective study was conducted in full compliance with the Declaration of Helsinki and was approved by the Ethical Committee of the University of Genoa (CERA 2024/79). Informed consent, covering the study’s purpose, procedure, risks, and benefits, was obtained from all the patients included. Additional signed releases were collected for the use of patient images and radiographies in the study.

A total of 50 patients, aged between 25 and 75 years (30 males and 20 females), who underwent immediate dental implant placement without restrictions of position and site (anterior and posterior teeth of the upper and lower jaw) concurrent with alveolar ridge preservation between January and July 2008, were included in the study. Only single-implant rehabilitations were included.

All patients were non-smokers and were treated at a private clinic in Acqui Terme, Italy. The primary outcome was bone level at each evaluated time point, while the secondary outcome was implant failure.

Exclusion criteria included the following:

- Smokers (regardless of the number of cigarettes/day);
- Chronic periodontitis individuals;
- Poor oral hygiene or non-compliance with care;
- Presence of systemic conditions, compromised medical states, or immunosuppressive conditions contraindicating elective surgery or affecting recovery (i.e., non-compensated diabetes);
- Use of anticoagulant medications or bisphosphonates;
- Pregnancy or lactation;
- Acute apical infections at the tooth planned for extraction.

All patients received tailored oral hygiene guidance and underwent professional mechanical plaque removal one week before surgery. The same specialist (NDA) performed all surgical and prosthodontic procedures.

**Figure 1:**
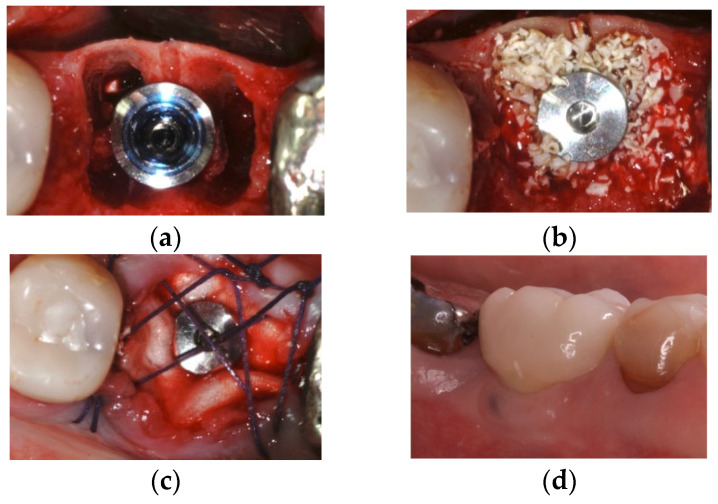
A case of immediate implant on lower molar: (**a**) implant placement, (**b**) marginal gap filled with hydroxyapatite, (**c**) the resorbable collagen membrane was left exposed and secured with horizontal mattress suture, and (**d**) final metal fused to porcelain cemented crown.

Patients received at least one implant immediately after the extraction, both in upper- and lower-anterior and -posterior sites. Surgical procedures were performed under local anesthesia using Articaine 2% with 1:100,000 epinephrine. An atraumatic extraction was performed, avoiding any damage to the buccal bone wall, with a minimal flap elevation in order to check the integrity of the buccal bone. The implants (Biomet 3i Full Osseotite Tapered and Straumann Bone Level) were inserted at least one millimeter under the bone level. Both implant systems are bone-level implants; Straumann^®^ implants (Straumann, Basel, Switzerland) have an SLA surface and cono-morse connection, while Biomet 3i^®^ (BIOMET 3i, Palm Beach, FL, USA) has an Osseotite surface and internal hexagon connection. The residual gap was filled with bovine hydroxyapatite (Bio-Oss Geistlich^®^, Geistlich, Switzerland) and then covered with a collagen membrane left exposed during the healing phase (Osseoguard regular, Biomet 3i^®^, Palm Beach, FL, USA), trimmed around the extraction socket, and secured with a cross-mattress resorbable suture (Vicryl Ethicon®, Somerville, NJ, USA) (Figure 1).

Antibiotic prophylaxis was administered as follows:

- Amoxicillin + Clavulanic Acid 875 + 125 mg, 2 g 1 h before surgery, or (in patients allergic to penicillin) Azithromycin 500 mg at the same timing
- Post-operative continuation with Amoxicillin + Clavulanic Acid 1 g 6 h after surgery or (in patients allergic to penicillin) Azithromycin 500 mg for 2 additional days

Non steroids anti-inflammatories (ibuprofen 600 mg) were left to the patient need and chlorhexidine 0.20% mouthrinses 3 times a day for 15 were requested on all the subjects.

One week after surgery, sutures were removed, and no temporary crowns or removable dentures were provided.

For the purpose of this investigation, only implants with X-rays at prosthetic delivery and 1 year after loading, 5 years after loading, and then 15 years after loading were included.

**Figure 2:**
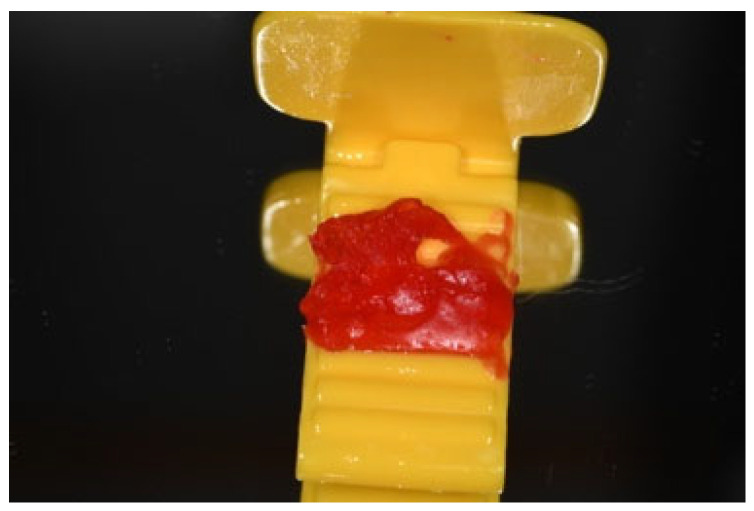
Customized film holder used for each time-point X-ray.

At all the steps, standardized intraoral radiographs were obtained with a parallel technique, using a personalized film holder with the customization of the bite (Figure 2).

Analogic X-rays were obtained using a De Gotzen^®^ (Varese, Italy) radiographic device, configured to operate at the minimum radiation dose compatible with diagnostic needs. This approach minimizes patient exposure while ensuring image adequacy. The analog radiographs were then digitally scanned using a high-quality flatbed scanner. The resolution was set at a minimum of 300 dpi (dots per inch) to ensure sufficient image detail for further analysis and digital processing. The images were saved in a lossless format (e.g., TIFF or PNG) to preserve their quality.

The digital images were imported into CorelDRAW Graphics Suite 2023^®^ for post-processing. Using the suite’s image adjustment tools (e.g., the Tone Curve, Brightness/Contrast, and Histogram Equalization functions), all radiographs were subjected to a standardized equalization protocol aimed at enhancing contrast in low-contrast areas, standardizing the grayscale across all images, and improving visibility of anatomical structures and pathologies. The equalization settings were applied uniformly to maintain consistency and reproducibility across the dataset. Measurements were carried out digitally with calibrated tools. This uniform method was applied across all datasets to enhance reproducibility and reliability.

Digital X-rays were obtained with CS 7200 Carestream System (Carestream Dental LLC, Atlanta, GA, USA) with an average setting of 0.180 mGray, following the same pattern as analogic ones. An independent examiner (E.C.) performed the final measurements 1, 5, and 15 years post-loading.

**Figure 3:**
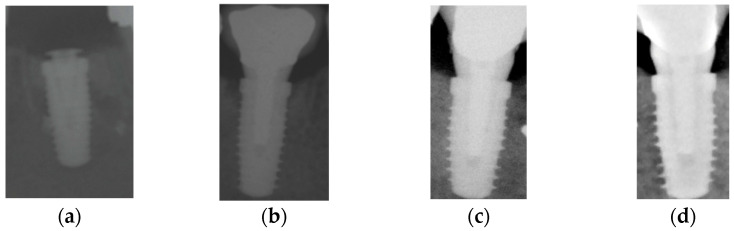
X-rays taken at each time point of the case displayed in Figure 1: (**a**) after implant placement, (**b**) 1 year after loading, (**c**) 5 years after loading, and (**d**) 15 years after loading. X-rays (**a**,**b**) were taken with analogical films and further digitalized.

Bone levels were measured by using the implant as reference, knowing the distance between the implant shoulder and the first thread, as well as the distance between the threads. In the case that both mesial and distal sides showed signs of resorption, only the deepest one was used for the purpose of this investigation. The primary outcome of the study was the evaluation of bone levels throughout the observation period (measurements were retrieved at each time point), while the secondary outcome was the number of implant failures (that is, the need to remove the implant) (Figure 3).

Three months after implant placement, definitive prostheses were delivered. All restorations were cemented and metal-fused to porcelain, and only the analogic impression technique was used.

Participants also received regular dental check-ups and professional oral hygiene treatments at six-month intervals during the follow-up period.

### 2.3. Statistical Analysis

Descriptive statistical analysis was used to obtain mean and standard deviations for bone levels (BLs) at all time points. In order to analyze the difference in mean BL at all the different time points, repeated-measurement analysis of variance was performed. The patient was the statistical unit for the analysis, and differences of means at the patient level were compared via *t*-test. For all tests, the level of significance was set at *p* < 0.005, and the confidence interval (CI) was 95%. The software used for the analysis was the IBM SPSS Statistics for iOS, Version 25.0 (IBM Corp., Armonk, NY, USA).

## 3. Results

Data from 50 patients were retrieved; 30 were male and 20 were female, with a mean age of 50 years, with a mean age of 49 and 51 years for women and men, respectively. A total of 20 implants were placed in the upper maxilla and 30 in the lower jaw; 30 implants were Biomet 3i Osseotite Tapered and 20 Straumann Bone Level, with a mean diameter of 4.0 mm and an average length of 11 mm.

Over the period of observation, all the implants were stable, they were in function at the last follow-up appointment (Table 1).

**Figure 4:**
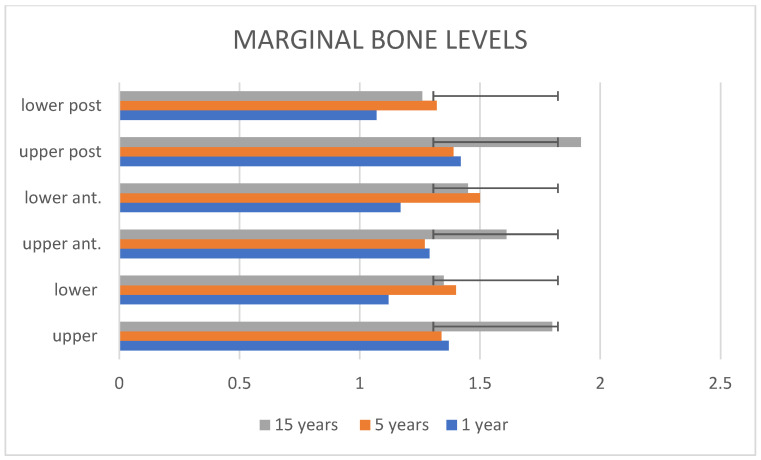
Schematic representation of the marginal bone-resorption levels divided into subgroups.

Bone-resorption levels showed a statistically significant difference between the upper and lower jaw one year after loading (mean, 1.37 ± 0.29 mm and 1.12 ± 0.19 mm, *p*-value 0.0015, respectively) and at the third time point at the 15-year check-up, where the maxillary elements exhibited a mean BL of 1.8 ± 0.38 mm, and the mandibular ones, 1.35 ± 0.27 mm (*p*-value 0.0000685). Anterior teeth, both upper and lower, were compared between them at each time point without statistically significant differences (Table 2). Posterior elements, both upper and lower, showed a statistical difference in bone-resorption levels at the first time point (1.42 ± 0.22 mm and 1.07 ± 0.2 mm, *p*-value 0.0001, respectively) and at the third observation (15 years) compared to anterior implants, where upper-posterior teeth exhibited a bone loss of 1.92 ± 0.35 mm, and lower-posterior teeth, 1.26 ± 0.22 mm (*p*-value 0.0001). No statistically significant differences were noticed at the second time point (1 year) (1.39 ± 0.24 mm for upper elements and 1.32 ± 0.26 mm for lower ones, *p*-value 0.39). No differences in bone-resorption levels were found between gender, nor were any connected to the age of the participants (Figure 4).

## 4. Discussion

The present retrospective study with a 15-year follow-up and involving 50 individuals provides data on the long-term performance of dental implants immediately placed in post-extraction sockets simultaneously with alveolar ridge preservation, particularly focusing on the changes in bone density across different regions of the jaw. No implant failures were recorded over the 15 years of follow-up, and mean bone measurements were satisfactory.

In a systematic review published in 2015 [14], where the mean follow-up period of the studies included was 13.4 years, cumulative mean values of survival percentages and mean marginal bone resorption were 94.6% and 1.3 mm, respectively, for immediate implants and simultaneous alveolar preservation. Considering the disparate outcome measures employed to assess dental implant performance, and within the limitations of this systematic review, it can be stated that immediate osseointegrated implants are safe and show high survival percentages, matching the results of the present retrospective study.

The study population consisted of 50 participants, 30 of whom were male and 20 female, with an average age of 50 years. The demographic distribution reflects a typical cohort for dental implant studies, where middle-aged adults often seek such treatments due to age-related tooth loss or trauma. The gender distribution, with a higher proportion of males, is also not uncommon in clinical studies of this type [15], though the implications of gender on the results were not directly addressed in the present study.

In this study, a total of 50 implants were placed, with 20 located in the upper maxilla and 30 in the lower jaw. This distribution suggests a greater demand for implants in the lower jaw [16]. Several factors could contribute to this, including differences in bone density and the biomechanics of occlusion; however, the number of subjects included in the present study is too small to draw conclusions. The distinction between implants in the upper and lower jaws is significant, as it provides context for understanding the different patterns of bone remodeling observed throughout the study [16].

When analyzing bone-level changes over time, the study offers a detailed examination at multiple time points, notably at one year post-loading and at a 15-year follow-up. These time points are crucial in assessing both the short-term and long-term behavior of the bone surrounding dental implants.

At the one-year post-loading mark, bone loss in the upper maxilla was 1.37 ± 0.29 mm, compared to 1.12 ± 0.19 mm in the lower jaw. This difference was statistically significant, with a *p*-value of 0.0015, indicating that bone loss in the upper jaw was slightly higher than in the lower jaw. This finding aligns with the general understanding that the upper jaw often exhibits greater bone loss due to its anatomical characteristics, such as softer bone quality and less dense trabecular structure, compared to the mandible [17,18].

By the 15-year follow-up, bone loss had increased in both regions. In the upper maxilla, bone loss was 1.8 ± 0.38 mm, while in the lower jaw, it was 1.35 ± 0.27 mm. The statistical significance of this difference was even more pronounced, with a *p*-value of 0.0000685. These data suggest that bone remodeling occurs at a different pace in the upper and lower jaws, with the upper maxilla experiencing more significant bone loss over the long term. The reasons for this could be multifaceted, including the anatomical differences between the maxilla and mandible, the type and distribution of occlusal forces, and potentially different biological responses to implant placement in these regions [19,20,21].

In addition to comparing overall bone levels between the upper and lower jaws, this study also differentiated between anterior (front) and posterior (back) implant sites in both regions. Interestingly, when anterior teeth were compared, no statistically significant differences in bone levels were observed between the upper and lower regions at any of the time points measured. This consistency suggests that implants placed in the anterior region, regardless of whether they are in the maxilla or mandible, may follow a similar pattern of bone remodeling. This could be due to several factors, including the generally lower occlusal forces on anterior teeth compared to posterior teeth, as well as the potentially more uniform bone quality in the anterior-jaw regions [15,22].

The analysis of posterior teeth, however, revealed a different outcome. At the one-year post-loading mark, a significant difference in bone loss was observed between the upper- and lower-posterior teeth. In the upper jaw, the posterior teeth showed a bone loss of 1.42 ± 0.22 mm, while in the lower jaw, bone loss was 1.07 ± 0.2 mm; the difference was statistically significant, with a *p*-value of 0.0001. This trend continued at the 15-year follow-up, where bone loss in upper-posterior teeth increased to 1.92 ± 0.35 mm compared to 1.26 ± 0.22 mm in the lower-posterior teeth, maintaining statistical significance with a *p*-value of 0.0001. However, at the second time point (which falls between the one-year and 15-year marks, 5 years), there was no significant difference between the upper- and lower-posterior teeth, with bone levels being relatively close (1.39 ± 0.24 mm for upper and 1.32 ± 0.26 mm for lower teeth, *p*-value of 0.39).

The greater bone loss observed in the posterior teeth, particularly in the upper jaw, could be attributed to several factors. Posterior teeth are subjected to higher occlusal forces during mastication, which can contribute to more pronounced bone remodeling and potential bone resorption around the implants. The anatomical characteristics of the posterior maxilla, including the presence of sinus cavities and softer bone quality, may also play a role in the greater bone loss observed in this region [23,24].

The clinical significance of these findings is multifaceted. For clinicians, understanding the differential behavior of bone loss in various regions of the jaw is crucial for planning implant treatments and managing patient expectations. The greater bone loss observed in the upper jaw, especially in posterior regions, suggests that more conservative loading protocols or adjunctive treatments, such as bone grafting, might be necessary to mitigate long-term bone loss and ensure the stability of the implants.

For patients, these results underscore the importance of ongoing maintenance and care following implant placement. Patients should be counseled on the potential for greater bone loss in the upper jaw and the need for regular check-ups to ensure the long-term health of their implants. Understanding these risks can help set realistic expectations and improve patient compliance with follow-up care, which is essential for the longevity of the implants.

Given the retrospective nature of the study and the inclusion criterion of a required radiographic follow-up at both 1.5 and 15 years, cases with early implant failure were inherently excluded. Therefore, no conclusions can be drawn regarding the survival rate of the implants, and such evaluation would require a prospective study including all implants from the time of placement.

Despite the valuable insights provided by this study, it also has some limitations that should be acknowledged. The sample size of 50 patients, while providing useful data, is relatively modest, and larger studies could provide more robust data and allow for subgroup analyses, such as examining the impact of age, gender, or systemic health conditions on implant outcomes. Additionally, while the 15-year follow-up is commendable and provides long-term data, even longer-term studies could offer further insights into the behavior of bone levels around implants as patients age and as the implants themselves continue to endure masticatory forces and other functional stresses. A possible limitation of the present investigation could be related to the radiographs, which were taken with two different systems (analogic for the T1 and digital at the other time points). It is worth mentioning that there is always a low risk of a not completely precise transfer of the pixels during the digitalization of the analogical films.

A further limitation of the present study is the use of two different implant systems with distinct surface treatments, which may have influenced the clinical and radiographic outcomes. Although previous studies have shown comparable long-term performance among different implant surfaces, this heterogeneity may introduce a potential bias in the interpretation of the results [25,26,27].

Another limitation of the present study is related to the number of variables, in particular, the position of the implants, which includes anterior and posterior teeth. In a systematic review, the authors highlighted the beneficial results achieved with immediate implants and alveolar ridge preservation for anterior and premolar teeth [23,24]; to the authors’ knowledge, there is no similar evidence regarding a comparison with posterior elements, but it seems reasonable to minimize surgical accesses, aiming to insert the implants at the time of the extraction, as described in a recently published article [28].

Ridge-preservation techniques reduce the horizontal bone changes which occur in the coronal portion of the buccal bone plate after tooth extraction when compared to spontaneous healing [29]. Moreover, as mentioned in this randomized clinical trial and applied in the present investigation, immediate implants should be placed slightly below the bone level (maximum 1 mm) to compensate for the spontaneous biological adjustment of the tissues after the healing phase.

Future research should also aim to explore other factors that may influence bone remodeling around immediate implants, such as the design and surface characteristics of the implants; the type of prosthetic restoration used; and patient-specific factors such as smoking status, systemic health conditions, and oral hygiene practices. These factors could all play a role in the long-term success of dental implants and are worth investigating in future studies.

## 5. Conclusions

This study provides valuable data on long-term bone-resorption levels around immediate dental implants placed in different sites of the lower and upper jaw simultaneously with alveolar ridge preservation. Significant differences in bone loss were found between anterior and posterior teeth, especially in the upper jaw, which might suggest carefully evaluating the need for immediate implants in the molar areas.

However, several limitations must be considered. First, the retrospective nature of the study prevents a full assessment of implant survival rates. Second, two different implant systems with distinct surface treatments were used, introducing potential bias in the interpretation of clinical outcomes. Lastly, the small sample size and the use of both analog and digital radiographic methods may affect the generalizability and consistency of measurements.

Future studies should incorporate standardized and validated radiographic protocols to further minimize variability, and ideally employ a prospective design with a larger cohort to enhance the robustness of findings.

## Figures and Tables

**Table 1:** Demographic characteristics of the sample of patients and distribution of dental implant position.

	Total
Males	30
Females	20
Age (total mean)	50
Age (women mean)	49
Age (men mean)	51
Implants in the upper jaw	20
Implant in the lower jaw	30
Biomet 3i	30
Straumann BL	20
Upper implants	20
Lower implants	30
Anterior implants	21
Posterior implants	29

**Table 2:** Mean bone-resorption levels at the 3 times interval.

	1 Year (mm)	*p*-Value (0.05)	5 Years(mm)	*p* Value (0.05)	15 Years (mm)	*p*-Value (0.05)
Upper n = 20	1.37 ± 0.29	0.0015	1.34 ± 0.33	0.503	1.8 ± 0.38	0.0000685
Lower n = 30	1.12 ± 0.19		1.4 ± 0.27		1.35 ± 0.27	
Upper ant. n = 8	1.29 ± 0.38	0.212	1.27 ± 0.45	0.213	1.61 ± 0.38	0.257
Lower ant. n = 13	1.17 ± 0.16		1.5 ± 0.24		1.45 ± 0.3	
Upper post. n = 12	1.42 ± 0.22	0.0001	1.39 ± 0.24	0.39	1.92 ± 0.35	0.0001
Lower post. n = 17	1.07 ± 0.2		1.32 ± 0.26		1.26 ± 0.22	

## Data Availability

The raw data supporting the conclusions of this article will be made available by the authors on request.
